# Supplementary material for: Splice-Junction-Based Mapping of Alternative Isoforms in the Human Proteome
Source: Cell Rep. Author manuscript; Available in PMC 2020 Jan 15. (PMC6961840; doi:10.1016/j.celrep.2019.11.026)
Supplement: 2 [file NIHMS1546469-supplement-2.zip › DF1/PXD009737/TestisLysC/TestisLysC_12_LGALS1_GGSLNLKPGECLRVRGEVAPDAK.pdf]

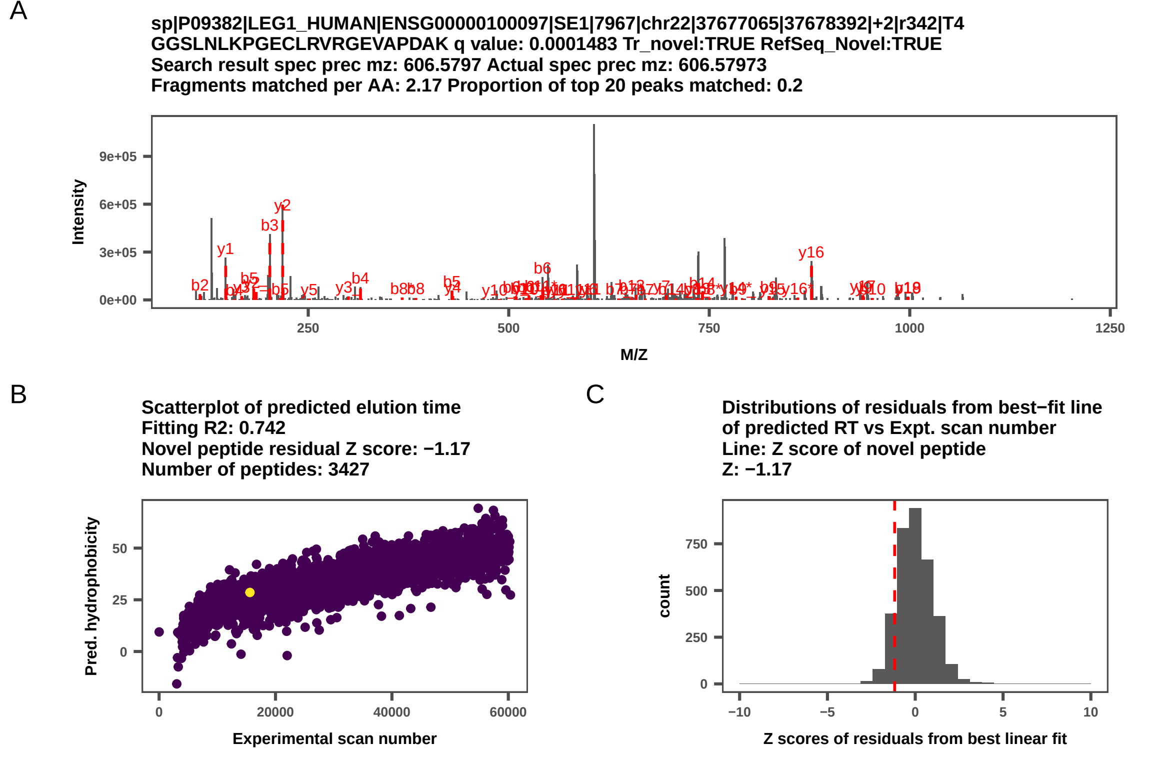
A
sp|P09382|LEG1_HUMAN|ENSG00000100097|SE1|7967|chr22|37677065|37678392|+2|r342|T4
GGSLNLKPGECLRVRGEVAPDAK q value: 0.0001483 Tr_novel:TRUE RefSeq_Novel:TRUE
Search result spec prec mz: 606.5797 Actual spec prec mz: 606.57973
Fragments matched per AA: 2.17 Proportion of top 20 peaks matched: 0.2
Intensity
9e+05
6e+05
3e+05
0e+00
y2
b3
y1
b2
b5
y5
y3
b4
b6
y16
M/Z
250
500
750
1000
1250
B
Scatterplot of predicted elution time
Fitting R2: 0.742
Novel peptide residual Z score: −1.17
Number of peptides: 3427
Pred. hydrophobicity
50
25
0
0
20000
40000
60000
Experimental scan number
C
Distributions of residuals from best−fit line
of predicted RT vs Expt. scan number
Line: Z score of novel peptide
Z: −1.17
count
750
500
250
0
−10
−5
0
5
10
Z scores of residuals from best linear fit
